# Development and implementation of digital peer mentoring in small groups for first-year medical students

**DOI:** 10.3205/zma001666

**Published:** 2024-02-15

**Authors:** Sabine Drossard, Anja Härtl

**Affiliations:** 1 University Hospital Augsburg, Department of Pediatric Surgery, Augsburg, Germany; 2 University Hospital Würzburg, Department of General, Visceral, Transplant, Vascular and Pediatric Surgery, Würzburg, Germany; 3 University of Augsburg, Medical Faculty, Medical Didactics and Educational Research, DEMEDA, Augsburg, Germany; 4 University Hospital Augsburg, Department of Hygiene and Environmental Medicine, Augsburg, Germany

**Keywords:** peer mentoring, digital mentoring, first year medical students, student mentors, professional identity formation, many-to-many, one-to-many

## Abstract

**Introduction:**

Mentoring has become an important educational strategy in medical training. Peer mentoring (PM) can enhance student experience and support transition to higher education. This article documents the implementation of an online peer mentoring program for first year medical students at a newly founded medical school in Germany during the COVID-19 pandemic and its development into in-person PM.

**Project description:**

We developed the program in close collaboration between students and teachers. Students were invited to apply as peer mentors via email; they received instructions and reflected on their role and experiences in meetings before, during and after the semester. One or more peer mentors were assigned randomly to a student group. We evaluated the program with an online survey inspired by the “Modified Mentorship Effectiveness Scale”. After successful piloting PM was implemented into the core curriculum.

**Results:**

In 2020 we assigned 17 peer mentors to 14 groups of 6-7 students. Groups met 3 or more times via Zoom^®^. Overall satisfaction was high. Both student groups reported benefits for their personal and professional identity formation. Atmosphere in online meetings was excellent. Most important topics were exams/learning strategies. In 2021 meetings were held in person. Overall satisfaction, perceived benefits and learning atmosphere were again rated very positively. Most students preferred many-to-many PM and random matching.

**Conclusion:**

The implementation of PM was successful and beneficial for the participating students. PM can help first-year medical students reduce anxiety, improve self-organization and orientation at university. It fosters identity formation and has positive effects on peer mentees as well as on peer mentors.

## Zusammenfassung

**Zielsetzung:**

Mentoring ist zu einer wichtigen didaktischen Strategie in der medizinischen Ausbildung geworden. Peer-Mentoring (PM) kann die Erfahrung der Studierenden verbessern und den Übergang in das Hochschulstudium unterstützen. Dieser Artikel beschreibt die Implementierung eines Online-Peer-Mentoring-Programms für Medizinstudierende im ersten Semester an einer neu gegründeten Medizinischen Fakultät in Deutschland während der COVID-19-Pandemie und dessen Weiterentwicklung als Präsenzveranstaltung.

**Projektbeschreibung:**

Das Programm wurde in enger Zusammenarbeit zwischen Studierenden und Lehrenden entwickelt. Die Studierenden wurden per E-Mail zur Teilnahme als Peer-Mentor*innen eingeladen; sie erhielten Instruktionen und reflektierten ihre Rolle und Erfahrungen in Treffen vor, während und nach dem Semester. Den Studierendengruppen des ersten Semesters wurden nach dem Zufallsprinzip ein oder mehrere Peer-Mentor*innen zugeteilt. Das Programm wurde mittels einer Online-Umfrage evaluiert, die sich an der „Modified Mentorship Effectiveness Scale“ orientiert. Nach erfolgreicher Pilotierung wurde das PM in das Kerncurriculum implementiert.

**Ergebnisse:**

Im Jahr 2020 wurden 14 Gruppen mit je 6-7 Studierenden insgesamt 17 Peer-Mentor*innen zugeteilt. Die Gruppen trafen sich mindestens dreimal im Semester über Zoom^®^. Die Gesamtzufriedenheit war hoch. Beide Gruppen, Peer-Mentor*innen und Peer-Mentees, berichteten von Vorteilen für ihre persönliche und professionelle Entwicklung. Die Atmosphäre in den Online-Meetings wurde als sehr gut bewertet. Die wichtigsten Themen waren Prüfungen und Lernstrategien. Im Jahr 2021 fanden die Treffen in Präsenz statt. Die Gesamtzufriedenheit, der wahrgenommene Nutzen und die Lernatmosphäre wurden erneut sehr positiv bewertet. Die meisten Studierenden bevorzugten PM in Gruppen („many-to-many“) und eine zufällige Einteilung ohne Matching.

**Schlussfolgerung:**

PM konnte erfolgreich in das Curriculum eingeführt werden und war für die Teilnehmenden von großem Nutzen. PM kann Medizinstudierende im ersten Semester dabei unterstützen, Ängste abzubauen sowie die Selbstorganisation und Orientierung beim Ankommen an der Universität verbessern. Es fördert die professionelle Identitätsbildung und hat positive Auswirkungen sowohl auf Peer-Mentees als auch auf Peer-Mentor*innen.

## 1. Introduction

Mentoring has become an important educational strategy in medical training all over the world, especially in North America and Europe [1]. Positive effects include enhancing personal and professional development for mentees [1], [2] as well as for mentors [3], [4]. Awareness of the benefits of mentoring in undergraduate medical education has increased [5], [6], [7]. Students and mentors may meet one-on-one or in a group setting (one-to-many or many-to-many). Mentors are assigned to students by student choice, different matching methods or randomly [1]. Some programs are formal with fixed meetings, some programs are informal or rely on social media only [8]. 

There has been a surge of mentoring programs for medical students in Germany in the last two decades: In 2000 Woessner et al. reported 10 German medical schools offering mentoring programs [9], while a more recent overview in 2011 found 22 mentoring programs offered by 20 of 36 German medical schools. Only 9 of them (41%) engage students as peer mentors [6]. While traditional mentoring programs rely on faculty members as mentors, lately peer mentoring (PM) programs with more experienced students mentoring younger students have been implemented in medical undergraduate training [10]. Relevant sources of anxiety for entry-level medical students include uncertainty on how to succeed in the local curriculum. Since students from higher semesters recently faced the same problems, they are uniquely qualified to support new medical students. Additionally, student peers may be perceived as more approachable than faculty members, lowering barriers for students and making it easier for them to speak openly [11], [12], [13]. PM can enhance student experience, overall wellbeing and support transition to higher education [11], [12], [14], [15], [16].

Various goals of PM programs have been described in other areas including academic, emotional, and social benefits [5], [10], [11], [17], [18], [19]. Published data on PM in medical schools is limited: studies found that PM provides psychosocial and academic benefits [13] and mentees felt “more prepared, supported and satisfied with their overall experience in medical school” [11]. A systematic review of medical school PM programs in 2018 found only 5 studies that met the inclusion criteria and identified three main outcomes: professional and personal development, stress reduction, and ease of transitioning [10]. Several studies have shown that peer mentors may also be affected by their participation in the program [11], [13]. 

Traditionally mentoring programs rely mainly on personal contact: In 2011 Meinel et al. found that regular contact between mentors and mentees was in 100% by personal meetings, additionally in 91% via mail and in 41% via phone. Digital meetings where not used to stay in contact [6]. There is no description of PM relying on digital meetings as of now. 

## 2. Project description

In consideration of the described challenges for first-semester medical students and the known benefits for mentees and peer-mentors, we developed a PM program for the newly founded Augsburg medical school to support students, promote personal and professional development, foster emotional well-being and establish a mentoring culture. The project was developed using the six-step Kern approach for medical curriculum development [20]. The following sections describe the six steps in detail. 

### 2.1. Problem identification and general needs assessment

Medical students experience high levels of stress and anxiety as they are exposed to a high academic workload, often in a competitive environment. They report higher stress levels than same-age peers in other academic environments [21], [22]. Studies consistently demonstrate a high frequency of psychological distress among medical students in various countries worldwide [23], [24]. Medical school training needs to prepare graduates for a professional medical career not only by providing skills and knowledge, but also by promoting professional and personal development and helping them adjust in their medical career [25]. Role models and mentors are described as some of the most powerful factors in professional identity formation [26], [27], [28] as well as “providing a welcoming community to facilitate the entry of students” [28]. 

### 2.2. Targeted needs assessment

In 2016 a new medical faculty was founded by the University of Augsburg. The first cohort of 84 students started their medical studies in Augsburg in winter semester 2019/2020. In summer semester 2020 a voluntary small-group mentoring program with faculty members was implemented (“Maturitas mentoring”). Students stated that they were missing advice and guidance from older students when navigating their medical studies. Due to the global pandemic summer semester 2020 was mostly conducted digitally. Lessons were only held in person if it was deemed absolute necessary. Therefore, Maturitas mentoring meetings were held via the online conference tool “Zoom^®^”. Medical students were experiencing unexpected disruptions of their studies as well as uncertainty regarding patient care and bedside teaching. This led to mental and emotional issues, stress, anxiety and sometimes fear [29], [30], [31]. Augsburg medical students also reported a decline in well-being in 2020 [32].

### 2.3. Goals and objectives

Considering the challenges posed by the pandemic in addition to the described conditions and findings from literature we developed a PM program for first-semester students at Augsburg medical school in addition to the existing faculty mentoring program.

According to the described theoretical framework, literature review and students’ comments the following aims of Maturitas PM were established:

- creating a positive culture of teaching and learning
- providing educational and emotional support for peer mentees
- reducing anxiety, stress und uncertainty when entering medical school
- fostering camaraderie and establishing student networks
- providing time and space to reflect on study-related issues
- supporting professional identity formation and sense of leadership in peer mentors

### 2.4. Educational strategies 

To encourage students to become actively involved in their education and process of professional identity formation, we used Co-creation, a close collaboration of students and teachers [33], [34], aiming to foster active engagement of students in the educational design of the PM program. 

 All second-year students were informed about the Maturitas PM program in August 2020 via E-mail and invited to participate. All first-year medical students starting in Augsburg in winter semester 2020/21 were included in the Maturitas PM program. Students were randomly divided into small groups of 6-7, deliberately ignoring the existing faculty group system so that PM groups differed from regular learning groups. Peer mentors were given the choice of working alone (one-to-many) or in a group (many-to-many). Groups and peer mentors were randomly assigned without matching methods. Three meetings with a duration of 90 minutes were scheduled for each group during the semester. Participation was voluntary. As a step towards student ownership, organization was delegated to the peer mentors. While the proposed dates were communicated as suggestions, peer mentors remained responsible for contacting their peer mentees, scheduling meetings, inviting their peer mentees and providing meeting links. All meetings were held digitally via Zoom^®^: students used free basic Zoom^®^ licenses and their own devices.

### 2.5. Implementation

The project was supervised by the Maturitas Team, which consisted of one doctor specializing in medical education, one clinical doctor with a focus on teaching and student assistants, providing organizational support. The project was supported by faculty stakeholders. 

Peer mentors received written information and were invited to online meetings. Organizational issues and educational questions were discussed in three online meetings that took approximately 120 minutes each and were held in October 2020, January 2021 and March 2021. In the first meeting students reflected on their role and responsibilities as peer mentors and discussed the aims of Maturitas PM as well as organizational aspects. They were informed about existing support offered by Augsburg university. Furthermore, peer mentors developed basic knowledge about group dynamics and different personalities, reflected on their own experiences in learning groups and discussed strategies for group moderation. The contact details of their mentees were communicated to the peer mentors, entrusting them with contacting their mentees and scheduling meetings. In the following meetings peer mentors shared and discussed their experiences, reflected on situations that occurred during the meetings and provided feedback. They reported the topics discussed during their meetings, duration and frequency of meetings, student participation and perceived difficulties and benefits.

### 2.6. Evaluation and feedback

The evaluation was conducted as online survey with EvaSys^®^ and sent to all students before and after the program. The questionnaire was designed in several steps by the Maturitas team and contained items assessing structure and organization of the meetings, perceived benefits of PM as well as students’ expectations and experiences. The survey was inspired by the “Modified Mentorship Effectiveness Scale” [13]. Meeting atmosphere was rated with items that were developed to evaluate Maturitas mentoring. Survey responses were recorded using a 5-point Likert scale. In addition, students were asked open-ended questions.

The pre-test and post-test were sent to participating students in the first week of and at the end of the semester. We extracted topics from oral and written feedback and clustered the open-ended questionnaire answers into categories. 

The following year the program continued as in-person mentoring. We used a shortened version of the online survey. Evaluation and feedback were used to iteratively revise and improve the program design, following the Kern cycle to ensure continuous improvement of the curriculum.

## 3. Results

In winter semester 2020/21 92 students began their medical studies at Augsburg University. 18 of 84 (21%) second-year students volunteered as peer mentors. Mentees and mentors were divided in 14 groups: 10 groups with individual peer mentors and 4 groups with mentor tandems. Evaluation participation (pre/post) was n=49 (52%)/n=25 (26%) for peer mentees and n=15 (83%)/n=11 (61%) for peer mentors. 

13 of 18 peer mentors attended the introductory meeting. We identified three main reasons to participate in the program: Professional support of peer mentees, emotional support of peer mentees and networking, social aspects and benefits for oneself. 11 of 18 peer mentors attended the second and third online meeting. Overall peer mentors felt that they were able to accommodate to their mentees’ needs. They gave positive feedback on the program and felt adequately prepared by the instructions they received, but wished for more information on group moderation and group dynamics. 

As reported by peer mentors during feedback sessions, the survey revealed that most groups met at least three times per semester, several groups meeting more often. Most peer mentees participated regularly. All meetings were held digitally via Zoom^®^. Online meetings were rated as sufficient substitutes for in-person meetings (4 or more points on the 5-point Likert scale) by 64% of peer mentees (M=3,52, SD=1,08, n=25) but only 22% of peer mentors (M=2,89, SD=1,27, n=9). The groups communicated via E-Mail and messenger services, some used online scheduling tools to schedule meetings. 

Peer mentees (4,52, SD 0,71) as well as peer mentors (4,45, SD 0,69) reported that the program met their expectations (5-point Likert scale, 5=strongly agree). Peer mentors were satisfied with their own performance (3,9, SD 0,94). Overall satisfaction and perceived benefits were high. Peer mentors rated the perceived benefits for themselves higher than peer mentees (see table 1 (Tab. 1)). 

Both student groups rated the atmosphere during PM meetings in winter semester 2020/21 very positively (see table 2 (Tab. 2)). Students from both groups reported that the first meeting was mostly directed by peer mentors but peer mentees increasingly influenced and structured the group meetings and provided their own topics. 

**Figure 1:**
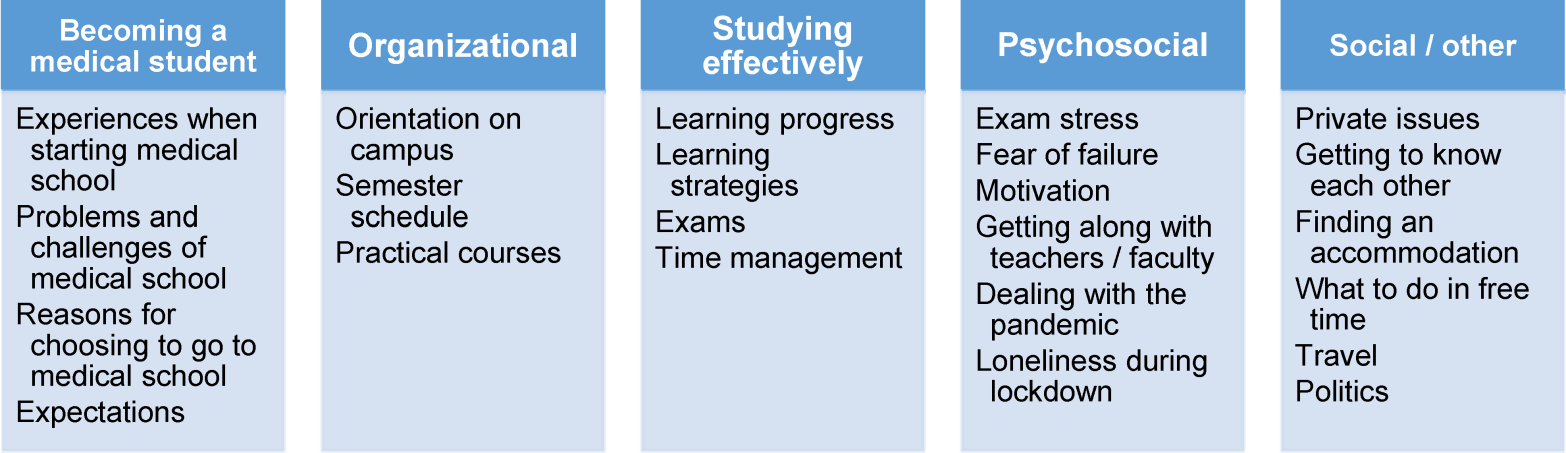
Topics during peer mentoring meetings as reported by students in open-ended questionnaire

Most prevalent topics were learning and exams, followed by organizational aspects (see figure 1 (Fig. 1)). Several aspects that students discussed were pandemic-related, like the feeling of isolation, dealing with social distancing, difficulty of motivating oneself when online learning, etc. 

**Figure 2:**
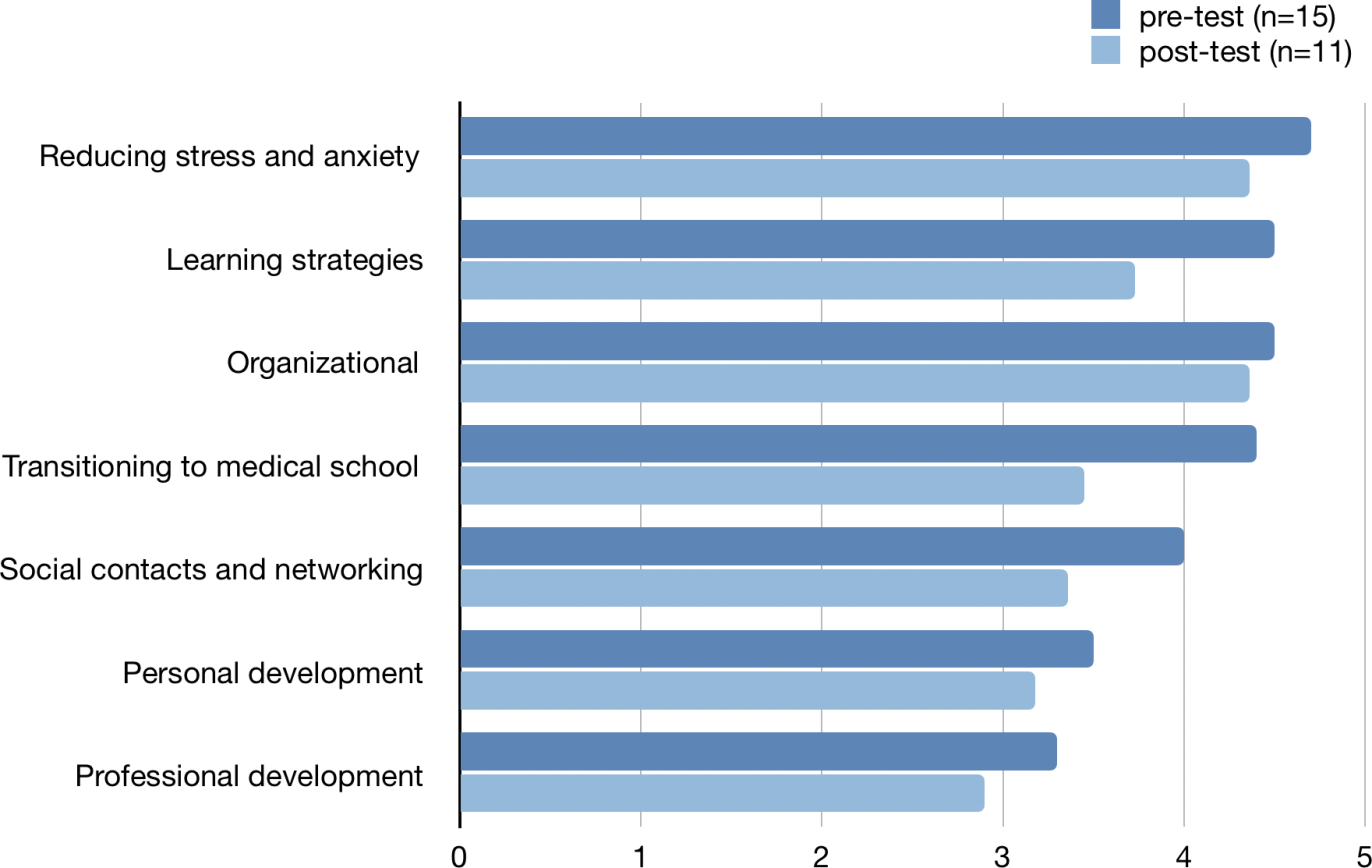
Pre-test: How much support do you think your peer mentees will need regarding the following aspects? Post-test: How much did you support your peer mentees in the following aspects? (5=very much, 1=not at all)

Peer mentors anticipated the needs of their peer mentees and have a good understanding of their needs and requirements. They reported organizational issues, reducing stress and anxiety and learning strategies as most relevant topics during meetings, followed by transitioning to medical school, social contacts/networking, personal development and professional development (see figure 2 (Fig. 2)).

Peer mentees rated their peer mentors as highly accessible, knowledgeable, approachable, supportive and encouraging. Peer mentors answered their questions satisfactorily and moderated the group sessions effectively. Students reported that their peer mentors helped them to adapt to their new role as medical students (see table 3 (Tab. 3)). 

## 4. Further development

After successful piloting PM was implemented into the Augsburg medical school curriculum. In winter semester 2021/22 33 second- and third-year students volunteered as peer mentors. 15 of 18 peer mentors (83%) decided to participate as peer mentor a second time. As proposed by students we established a cooperation with the student council (“Fachschaft”), grouping PM groups in the student council’s introductory week. 89 students were assigned to 15 groups: 12 groups of 5-6 peer mentees with 2 peer mentors and 3 groups of 7-9 peer mentees and 3 peer mentors. Three meetings were scheduled for each group. Group discussion and introductory meetings of the Maturitas team and peer mentors were expanded to meet students’ demand. Two introductory and training meetings (2 and 3 hours) were held before and 3 meetings for feedback and exchange during and after the semester (1-1.5 hours each). Training methods and contents remained constant but were expanded.

The evaluation questionnaire was adapted and shortened. We asked students what group size and mentor-to-mentee ratio they would prefer as well as their preferred duration of the program. Furthermore, we asked peer mentors to elaborate on their experiences in contrast to online PM. 14 peer mentors (42,4%) and 27 peer mentees (30,3%) completed the questionnaire. 

Overall satisfaction was high (see table 1 (Tab. 1)). Both student groups reported that the program met their expectations (Mentees: 4,33, SD 0,78. Mentors: 4,07, SD 1,07. 5-point Likert scale, 5=strongly agree). Learning climate was considered excellent (see table 2 (Tab. 2)). Mentors were satisfied with their own performance (4,14, SD 0,36. 5-point Likert scale, 5=strongly agree). 

85,7% of peer mentors and 65,4% of peer mentees were motivated to participate as peer mentors next year. 85,7% of peer mentors and 81,4% of peer mentees preferred mentor tandems. Group size was considered ideal by 85,7% of peer mentors and 92,5% of peer mentees. Most students preferred random grouping of mentees and free selection of mentor partner over matching methods. 67% of peer mentees and 43% of peer mentors preferred the program to continue over two semesters. Peer mentors stated that in-person meetings were more personal and open and that it was easier to involve every member of the group in the conversation. Students felt closer bonds to each other when meeting in person as opposed to digital meetings, but they also stated that digital meetings were easier to schedule and they had met more often in winter semester 2020/21. 

## 5. Discussion

Implementing a PM program at a newly founded medical school during a pandemic was challenging but possible. Our educational goals were achieved in a highly participatory approach for the design, implementation and further development of the program. 

The reported results and students’ statements show that the intended goals of the program where met. Both student groups reported that the program met their expectations. Results of our evaluation were in agreement with previous studies conducted in regards of overall satisfaction and perceived benefits [10], [11], [13], [18], [19]. No group finished the program early and meeting attention was high, even though participation was voluntary. As seen in other peer mentoring programs, our students met more often than scheduled by the program [18], [19].

Peer mentees rated their peer mentors as highly effective in guiding them and providing feedback. They were satisfied with the educational and psychosocial support they received. 

Our peer mentors were suitable sources of advice for the following medical students. Students were able to discuss topics that were important to them. The areas of support reported by our students are congruent with previous project reports that list professional and personal development, stress reduction, and ease of transitioning [10] as well as anxiety reduction, social support and more effective studying [13] as central aspects of their program.

At the newly founded medical school only a limited number of students were available, restricting the development of a matching system. We assigned students randomly. Since peer mentees rated their peer mentors as very effective, we believe that no matching may be required for PM programs, disagreeing with Altonji et al. [13]. This may be attributable to the fact that our program focuses on psychosocial support rather than academic promotion. 

In our program peer mentors worked alone, in tandems or in groups of 3, which all students found useful. Meinel et al. [6] found only 3 of 22 mentoring programs at German medical schools that offer many-to-many group mentoring. Our students preferred peer mentor tandems over single peer mentors. We therefore recommend considering the implementation of many-to-many PM programs. 

As reported before [10], [11], not only peer mentees as the primary target group but also peer mentors benefitted from the program. Peer mentors practiced their role as role models and teachers for younger students, learned about group moderation skills and took on responsibility for their peer mentees, thus fostering their own personal and professional development. Students participating in the program benefitted by creating a social network with their fellow students from different years. 

Following the co-creation strategy for the development of Maturitas PM turned out to be useful, as the program is highly accepted by students. We recommend involving students in the development of mentoring programs.

To our knowledge this is the first report of digital PM for first-year medical students. Until now mentoring has been a domain of personal meetings, limiting its application. Online meetings provided a good learning environment. Students felt that they were able to speak openly, participate actively and have positive and appreciating conversations even when the groups did not meet in person. The successful implementation of digital PM in small group discussions via an online conference tool can act as an example for other situations in which in-person mentoring is not feasible due to remote locations of students, multiple sites where learning takes place or other limitations.

### Limitations

Summer semester 2020 at Augsburg medical school was held exclusively online, so Augsburg medical students were experienced in online learning as well as online mentoring and adept at using Zoom^®^. Therefore, our experiences may not be transferable to other local situations. 

The survey consists of subjective assessments. No objective evaluation was made, so not all effects of the program are quantifiable. Since meetings in winter semester 2020/21 were only held digitally and during a digital semester while in-person meetings in 2021/22 were embedded in face-to-face classes, a comparison of online meetings vs. in-person PM meetings is not feasible.

## 6. Conclusion

Digital PM was implemented successfully at a newly founded medical school during the global pandemic and contributed to the building of student networks, supporting first-year medical students despite widespread online teaching. PM can help first-year medical students to reduce anxiety, improve self-organization and foster identity formation in their new role as medical students. PM can support students’ orientation at university and has positive effects on peer mentees as well as peer mentors. Online PM is feasible even in situations with limited means. In group online meetings an open and trusting conversation atmosphere can be established. Our students preferred many-to-many mentoring and random grouping. We propose to include students as early as possible when developing mentoring programs to adapt them to students’ needs.

## Authors’ ORCIDs

- Sabine Drossard: 0000-0002-3442-4851
- Anja Härtl: 0009-0008-0818-6213

## Acknowledgement

We would like to thank all students who supported this project and actively contributed to its conduct and development. Furthermore, we would like to thank the management of Augsburg Medical Faculty, the Department of Medical Education and the Head of Pediatric Surgery who supported its implementation.

## Competing interests

The authors declare that they have no competing interests. 

## Figures and Tables

**Table 1:**
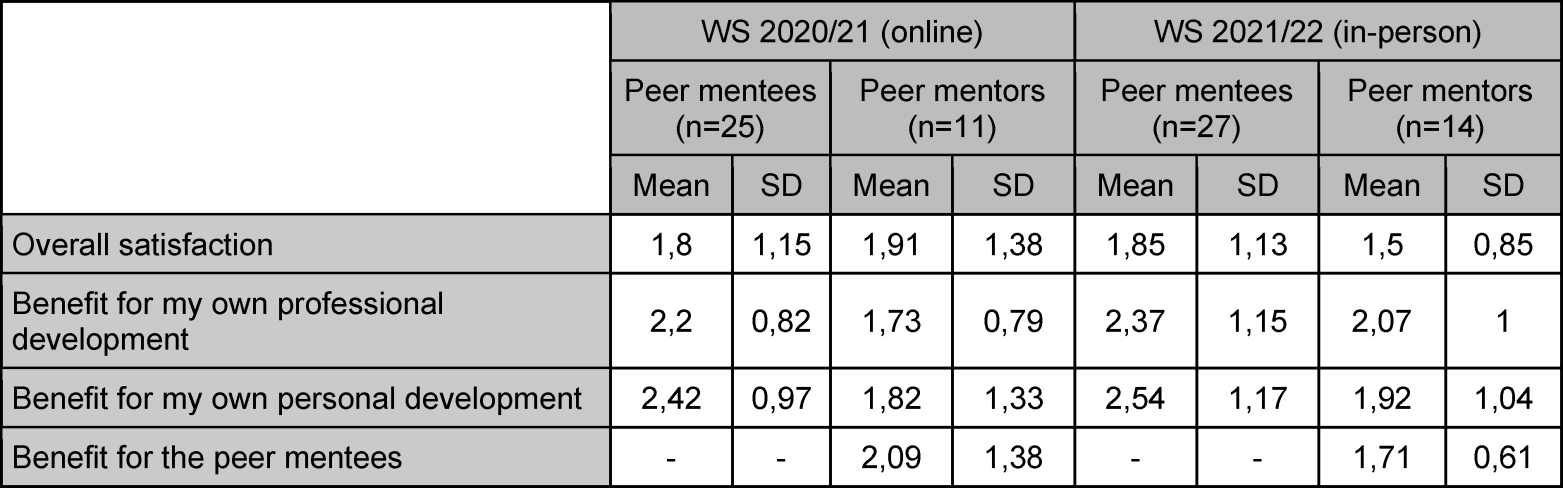
Overall satisfaction and perceived benefits as reported by both student groups on a 5-point Likert scale (1=excellent, 5=poor)

**Table 2:**
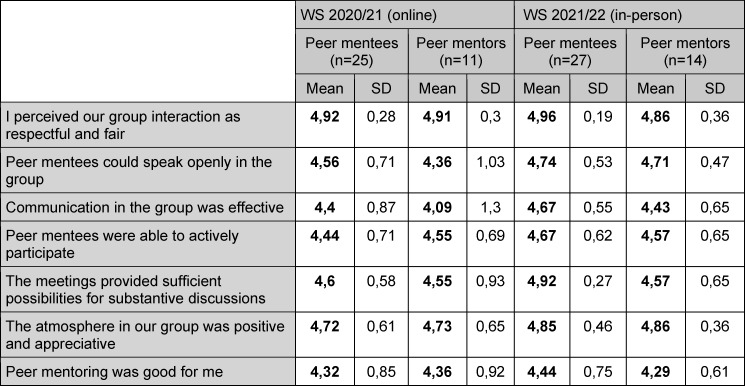
Rating of learning atmosphere in winter semester 2020/21 (online) and 2021/22 (in-person). 5-point likert scale (1=strongly disagree, 5=strongly agree)

**Table 3:**
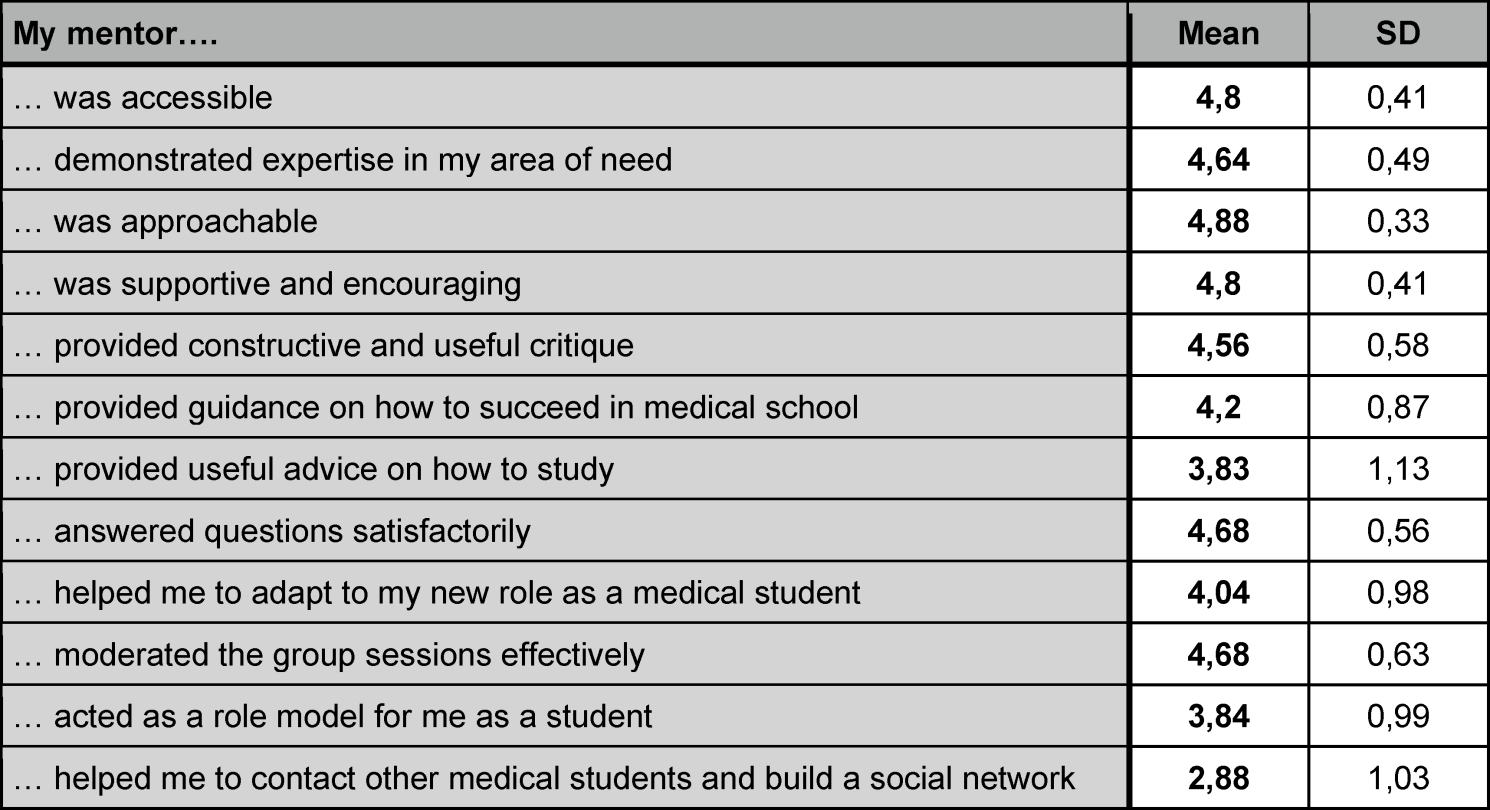
Rating of peer mentor effectiveness by peer mentees in winter semester 2020/21. 5-point Likert scale (1=strongly disagree, 5=strongly agree), n=25
